# Current Perspectives on JC Polyomavirus Transmission and Associated Diseases: Implications for Prevention in Risk Populations

**DOI:** 10.3390/v18070716

**Published:** 2026-06-29

**Authors:** Joana M Oliveira, Cristina Luxo, Ana M Matos

**Affiliations:** 1 CERES—Chemical Engineering and Renewable Resources for Sustainability, Faculty of Pharmacy, University of Coimbra, 3000-548 Coimbra, Portugal; joana.oliveira@student.ff.uc.pt (J.M.O.); crisluxo@ci.uc.pt (C.L.); 2 Centre for Functional Ecology (CFE), Associate Laboratory TERRA, Department of Life Sciences, University of Coimbra, 3000-504 Coimbra, Portugal; 3 Laboratory of Microbiology, Faculty of Pharmacy, University of Coimbra, 3000-548 Coimbra, Portugal; 4 Laboratory of Clinical Analysis of University of Coimbra, 3004-504 Coimbra, Portugal

**Keywords:** JC polyomavirus, transmission, PML

## Abstract

JC polyomavirus (JCPyV) is a worldwide-distributed human virus. Primary infection with JCPyV is usually asymptomatic and followed by a lifelong persistent infection. In situations of profound immunosuppression or prolonged treatment with specific immunomodulatory drugs, such as natalizumab, viral reactivation can occur and lead to the development of JCPyV-associated diseases. Progressive multifocal leukoencephalopathy (PML), a severe demyelinating disease of the central nervous system, is the most common clinical manifestation of JCPyV reactivation. Less frequently, viral reactivation may be associated with granule cell neuronopathy, encephalopathy, and meningitis. However, the pathogenesis of these diseases remains a subject of debate. To date, no treatment is available for JCPyV infection. Nevertheless, some therapeutic options have been explored. Despite its ubiquity, the main mode of JCPyV transmission remains unclear. Epidemiological data suggests that primary infection may be acquired in childhood and throughout life, with the involvement of different routes of transmission. In the absence of an effective treatment, the prevention of infection is crucial in risk groups, such as immunosuppressed or natalizumab-treated patients. Therefore, until the achievement of an effective antiviral molecule or a prophylactic vaccine, prevention measures will rely on avoiding transmission, for which it is crucial to understand how transmission occurs. The present review emphasizes the current data on JCPyV transmission routes and associated diseases, including pathogenesis, diagnosis and potential treatment options, highlighting the importance of further studies.

## 1. Introduction

JC polyomavirus (JCPyV) is a non-enveloped human virus with a double-stranded circular DNA (dsDNA) genome comprised within an icosahedral capsid [1,2]. In 1971, this polyomavirus was first identified in the brain of a patient with Progressive Multifocal Leukoencephalopathy (PML) [3].

Primary infection with JCPyV is usually asymptomatic and followed by a lifelong persistent asymptomatic infection. In cases of severe immunosuppression, such as that caused by human immunodeficiency virus (HIV) infection or prolonged treatment with immunomodulatory drugs, particularly natalizumab, JCPyV reactivation may occur and lead to the development of JCPyV-associated diseases [1,4]. The most common clinical manifestation of JCPyV reactivation is PML, a rare and often fatal demyelinating disease of the central nervous system (CNS) [3]. Although less common, viral reactivation can also result in granule cell neuronopathy, encephalopathy and meningitis. The underlying pathogenesis of these diseases remains a subject of debate [5]. Currently, no treatment exists for JCPyV infection. Nevertheless, some therapeutic strategies, including the use of JCPyV-specific T cells, have been explored [4,6].

JCPyV is widely distributed among the human population, with seroprevalence values ranging from 34% to 91% [7,8,9,10,11,12,13,14]. These values vary according to the geographic region; demographic characteristics of the studied population, especially age; and assay methodologies applied [2] (Table 1). The majority of seroprevalence studies exclude children, which could introduce bias into conclusions regarding the seroprevalence profile of this virus [15,16].

A recent systematic review and meta-analysis considering exclusively seroprevalence studies on healthy individuals identified high seroprevalence values for young children and older individuals (over 50 years old), with a decline in late childhood and early adulthood [17]. This seroprevalence profile suggests that primary infection occurs in early childhood, after which the virus establishes a persistent asymptomatic infection, and, consequently, antibodies may wane. In older individuals, an increase in antibodies is probably due to viral reactivation or new exposure to the virus and the consequent stimulation of the immune system [11,15,17].

The main mode of JCPyV transmission remains to be defined. Considering the ubiquity of JCPyV and its detection in various types of samples, multiple routes of transmission may be involved [18,19]. The presence of this virus in respiratory samples of children points to the possible implication of respiratory transmission. Its detection in urine, stool and some environmental samples may suggest the involvement of the fecal–oral route [7,19,20,21,22]. Moreover, JCPyV has been identified in the vaginal secretions of non-pregnant women and in semen samples [23,24,25], which suggests the potential implication of sexual transmission.

The present review provides an overview of the available data on potential JCPyV transmission routes, associated diseases, pathogenesis, diagnosis and available therapeutic options.

**Table 1:** Seroprevalence of JCPyV according to demographic characteristics of the studied population and geographic region.

Studied Population	Age Range (Years)	Country	Seroprevalence Value	Ref.
Healthy individuals	1–70+	USA	39–77%	[16,26,27]
Healthy individuals	0–94	Taiwan	63%	[28]
Healthy individuals	50+	China	81%	[29]
Healthy individuals	26–76	Australia	63%	[13]
Healthy individuals	4	Greece	34%	[30]
Healthy individuals	20–59	Switzerland	58%	[31]
Healthy individuals	25–75	Portugal	91%	[7]
Healthy individuals	1–93	Italy	43–72%	[10,15,32]
Healthy individuals	1–64	Czech Republic	57%	[33]
Healthy individuals	14–50+	The Netherlands	63%	[14]
Healthy individuals	0–50	Finland	45–69%	[11,34]
HIV-patients	25–75	Portugal	91%	[7]
Multiple sclerosis patients	<30–50+	Spain	55%	[9]
Multiple sclerosis patients	18–74	Portugal	61%	[8]
Patients with lung cancer	50+	China	73%	[29]
Patients with cutaneous squamous cell carcinoma	35–85	USA	88%	[27]
Non-Hodgkin lymphoma patients	20–74	USA	49%	[35]
Patients with neuroinvasive diseases of unknown human etiology	9–93	Croatia	89%	[12]

## 2. JCPyV Structure and Genome

JCPyV is a non-enveloped virus with a supercoiled, covalently closed dsDNA genome of approximately 5000 base pairs (bps). The viral genome comprises early and late coding regions, separated by a highly variable non-coding control region (NCCR) [36,37].

Transcription of the viral genome occurs bidirectionally from the NCCR. The early coding region encodes the tumor antigens, small (t) and large (T), which are responsible for initiating viral replication [36]. These early proteins are essential to increase viral transcription by working as a helicase to unwind the supercoiled dsDNA and as an enhancer for recruiting host cell machinery. Also, among other functions, t-antigen interrupts the cytokine signaling pathway of the innate immune response, and T-antigen inactivates tumor suppressor proteins, including p53 [4,38].

The late region encodes the agnoprotein, a small, non-structural regulatory protein, whose functions include the regulation of viral transcription and deregulation of host cell DNA repair mechanisms [39,40]. The expression of this protein is essential to a productive JCPyV life cycle due to its interaction with a large number of cellular proteins and organelles [41]. For instance, results from “in vitro” studies have described the interaction between agnoprotein and the mitochondria of glial cells in culture. Some of these cell lines were able to differentiate into oligodendrocytes when submitted to appropriate growth conditions, which allowed the authors to explore the negative impact of agnoprotein on this process. Based on the obtained results, it has been hypothesized that agnoprotein negatively affects energy production, ultimately promoting apoptosis [42]. Further, to promote viral dissemination, this protein leaves the host cell prior to its death and interacts with adjacent cells. Given its important role, deletion of the agnoprotein gene results in a virus that is unable to propagate [4,41,43].

The late coding region also encodes the structural proteins VP1, VP2 and VP3, which are crucial for capsid formation. [44,45]. Despite being the major structural protein of the viral capsid, VP1 requires minor proteins VP2 and VP3 for stabilization [4]. As JCPyV is a non-enveloped virus, VP1 binds directly to sialic acid residues on the host cell membrane to induce endocytosis. Also, this structural protein interacts with serotonin receptors, especially 5-HT_2A_, for further internalization of the virus [44,46].

The genetic diversity of VP1 nucleotide sequences allows JCPyV to be classified into different genotypes and subtypes. Although JCPyV is found worldwide, its viral genotypes and subtypes vary according to geographic regions. Genotype 1 was first related to Europe and America, genotypes 2 and 7 to Asia, genotypes 3 and 6 to Africa, and genotype 8 to the Western Pacific [2,7,47,48]. Nowadays, due to human dispersal throughout the world, other genotypes have been frequently detected in these continents. For instance, genotypes 2 and 4 have been frequently reported in European countries [49].

Furthermore, some authors have reported different genotypes in healthy individuals and immunocompromised patients, particularly those infected with HIV [7,50,51]. This suggests that alterations in VP1 may be linked to the pathogenesis of the virus [49]. For instance, in Portugal, types 1B, 4, and 2B were more frequently observed among healthy individuals, while types 1B, 2B, and 3 were more frequent in HIV-infected patients [7]. In Italy, genotype 2 was more frequently detected among patients treated with immunomodulatory therapies [52].

Between the early and late coding regions, the JCPyV genome contains a hypervariable NCCR. This region includes regulatory elements, including promoters, enhancers, and silencers, as well as the origin of replication [1,36]. The sequence and structure of this region allow for the classification of JCPyV strains as archetype or rearranged [1,36].

The archetype strain of the JCPyV presents a conserved NCCR, which includes six boxes (A, B, C, D, E and F) [1,53,54,55]. This strain is considered to be the transmissible form, usually found in the environment, and is associated with a lifelong persistent asymptomatic infection [56,57].

The JCPyV rearranged strains exhibit an NCCR with deletions, duplications or substitutions. The most common rearrangement is the deletion of the D box, which has been identified as an important rearrangement for the development of CNS pathologies. Mad-1 is the most studied rearranged strain of JCPyV and is characterized by the deletion of the B and D boxes, as well as repeats of the A, C and E boxes (two 98 bp tandem repeats) [1,53,54,55]. JCPyV-associated diseases have been linked to rearranged virus strains [56,57].

Although multiple studies have been performed to understand the implications of viral proteins on the replication of JCPyV, particularly their interactions with host cell machinery, several questions remain to be clarified, and further studies are required.

## 3. JCPyV Transmission

The main mode of JCPyV transmission remains unclear. However, due to its high seroprevalence, simple and common routes of transmission have been suggested, such as respiratory and fecal–oral modes [18,58].

### 3.1. Respiratory Transmission Hypothesis

Tonsillar tissue has been identified as the initial site of JCPyV infection, which suggests that viral particles could be transmitted through the upper respiratory system [59,60]. Thus, some studies have been performed to assess the detection of this virus genome in various types of respiratory samples [18,61,62,63,64,65].

JCPyV has been rarely reported in saliva and oropharyngeal washing samples [61,64]. Nevertheless, these results may be related to limitations of the study design, namely, the type of sample used, the restricted sample size and the age range of the included individuals [61,63,64]. Although primary infection appears to occur during the first years of life, the majority of these studies have not included young children [61,62,63,64,65]. In a recent study addressing some of these limitations, the JCPyV genome was detected in nasopharyngeal secretions of children, particularly those under six years old [18]. The results obtained support the hypothesis that primary infection may occur in the first years of life and that respiratory secretions may be implicated in JCPyV transmission.

Further studies overcoming the mentioned limitations are required to fully understand the implication of the respiratory route in JCPyV transmission.

### 3.2. Fecal–Oral Transmission Hypothesis

After primary infection, epidemiological data points to the establishment of a persistent JCPyV infection in the kidneys, with low levels of viral replication resulting in a continuous asymptomatic urinary excretion [58,66]. In fact, JCPyV is frequently detected in urine samples of both immunocompetent and immunosuppressed individuals [7,20,67]. Furthermore, some studies have reported the presence of this virus in stool samples from adults [20,68]. As expected, due to such an excretion profile, JCPyV has been repeatedly detected in sewage systems worldwide, including in Portugal, Spain, Sweden, France, Latin America, Greece, Egypt, the USA and Australia, regardless of the time of the year [19,58]. This polyomavirus has been detected in both untreated and treated wastewaters, which suggests that treatment processes applied in wastewater treatment plants are not efficient in removing viruses [19,69].

Treated wastewaters are discharged into the environment and could represent a potential source of viral particles [22,70]. In fact, environmental matrices, including wastewaters, are important reservoirs for human viruses, especially for non-enveloped viruses with a double-stranded DNA genome. Viruses with these features, such as JCPyV, are more resistant to environmental stress, including temperature and UV light [69,70].

Treated wastewaters can be used in irrigation systems and contaminate fresh vegetables and soft fruits [19,21,71], which could be ingested by humans. Moreover, bivalves, organisms with a natural biofilter behavior, can accumulate and concentrate viruses present in polluted waters [21,70]. In fact, the JCPyV genome has been detected in bivalves, including oysters and mussels, and was already identified in a strawberry sample [21,22]. The presence of the viral genome in these types of samples inevitably leads to questions regarding the virus’s infectivity. However, a recent study demonstrated a correlation between the presence of JCPyV genome and infectious viral particles in wastewater samples, using the non-enveloped Mengo virus [72], supporting the hypothesis that even if only viral genome was evaluated, its presence may be linked to the presence of infectious viral particles. These findings, along with the structural characteristics of JCPyV, suggest that contaminated water and food may be important vehicles for JCPyV transmission through the environment, namely, through the fecal–oral route [4].

During the SARS-CoV-2 pandemic, the rate of JCPyV seroconversion remained stable in regions where the use of masks was mandatory, even though the rate of seroconversion of known respiratory viruses (e.g., influenza virus) declined significantly [73,74]. Despite the limited data available, it appears that the implementation of restrictive measures to prevent the respiratory transmission of infectious agents did not have a significant impact on new infections with JCPyV. These data suggest that JCPyV infection could also occur through another route of transmission. Given the high seroprevalence of the virus, it can be hypothesized that, in addition to the respiratory route, another common route of transmission, such as the fecal–oral route, should also be considered.

Nevertheless, further research would help clarify the importance of the fecal–oral route in JCPyV transmission.

### 3.3. Other Routes of Transmission

As occurs for other viruses, multiple routes may be involved in the transmission of JCPyV, such as the vertical mode. Nevertheless, studies exploring the implication of this route in the transmission of JCPyV are extremely limited and report conflicting results [75,76,77,78].

In 1998, the hypothesis of JCPyV transplacental transmission was first studied using samples of placenta, brain and kidney tissues of aborted fetuses. However, this virus was not detected in any of the analyzed samples [75]. Years later, another study assessing JCPyV transplacental transmission with samples of umbilical cord blood also rendered negative results [77]. Despite the absence of JCPyV in those types of samples, Boldorini and colleagues (2011) reported serological evidence of JCPyV vertical transmission, suggesting a potential mother-to-newborn transmission soon after birth [76]. These results were supported by the findings of Mazzoni and colleagues (2020), who reported serological evidence and the detection of JCPyV in placenta and umbilical cord blood samples [78].

Nevertheless, considering the limited number of studies, further investigations using other types of samples, such as amniotic fluid, are needed to clarify the implication of the vertical route in the transmission of JCPyV.

The sexual route is a well-documented mode of transmission for multiple microorganisms. Although limited, recent studies have been focused on the hypothesis of the sexual transmission of JCPyV. This virus has been detected in semen samples of both fertile and infertile men, as well as in vaginal secretions from non-pregnant and pregnant women [23,24,25,78]. While scarce, these preliminary data suggest the possible implication of these secretions in the transmission of JCPyV. Nevertheless, further studies should be performed assessing the significance of this route in JCPyV transmission.

## 4. JCPyV Infection and Associated Diseases

**Figure 1:**
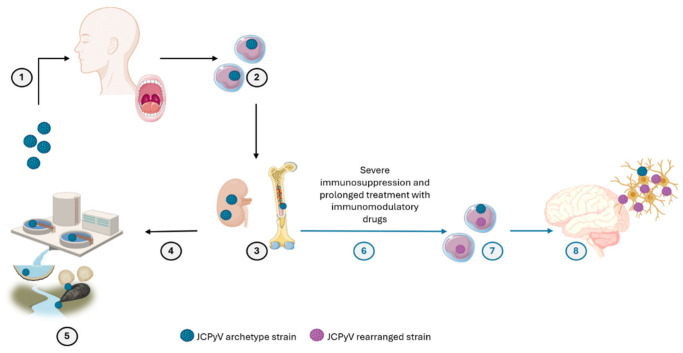
Schematic diagram of infection with JCPyV. The figure illustrates events thought to occur during infection with JCPyV. **1**: Asymptomatic primary infection with archetype JCPyV strain on tonsillar tissue; **2**: infection of B-lymphocytes with JCPyV archetype strains; **3**: persistent asymptomatic infection, possibly in kidney and bone marrow; **4**: asymptomatic urinary excretion of JCPyV archetype strains due to low levels of viral replication in the kidney; **5**: presence of JCPyV archetype strains in different environmental matrices (such as wastewaters and bivalves, which may function as sources of viral particles for human infection); **6**: severe immunosuppression or prolonged treatment with immunomodulatory drugs, such as natalizumab, may result in active viral replication and emergence of a neuropathogenic rearranged JCPyV strain; **7**: B-lymphocytes infected with JCPyV rearranged strains reach the CNS; **8**: development of PML and other rare CNS-associated JCPyV diseases.

JCPyV infection is associated with an asymptomatic primary infection, likely occurring in the tonsillar tissue. Following primary infection, this virus establishes a persistent asymptomatic infection, probably in the kidney, bone marrow and lymphoid tissues [4,6]. In situations of profound immunosuppression, particularly related to HIV infection, hematological malignancies, or the use of immunosuppressive drugs, viral reactivation may occur and lead to the development of JCPyV-associated diseases [56] (Figure 1).

### 4.1. Clinical Manifestations

The most common clinical manifestation of JCPyV reactivation is PML, a rare and often fatal demyelinating disease of the CNS [57]. Interestingly, the ubiquity of JCPyV contrasts with the rarity of PML, even among immunocompromised individuals [6]. Such a discrepancy may be related to the different cellular tropism of different strains of JCPyV. PML results from the lytic JCPyV infection of oligodendrocytes. Nevertheless, the neurotropism of JCPyV depends on rearrangements in its NCCR, which may result in a replicative advantage and neurotropism [79]. During viral persistence and associated replication, several rearrangements occur; however, most of these produce non-viable viruses. The rearrangements responsible for conferring new tissue tropism and pathogenic potential are very limited, which may explain the rarity of PML disease [79].

The pathogenesis of PML, particularly how JCPyV rearranged strains reach the CNS, remains to be clarified. A hypothesis is the entry of the rearranged strains through infected lymphocytes or the reactivation of JCPyV archetype strains already present in the CNS [3,5,6,80] (Figure 1). The blood–brain barrier is effective in preventing the spread of JCPyV, but even temporary immunosuppression can allow virus infiltration by B cells [4].

In addition to PML, the reactivation of JCPyV has been suggested as the cause of other, less frequent CNS diseases, namely, JCPyV granule cell neuronopathy, JCPyV encephalopathy and JCPyV meningitis [3].

#### 4.1.1. Classic PML

PML was first described in 1958 as a complication of primary B cell lymphoproliferative diseases [79]. Indeed, hematological malignancies still represent an important risk factor for the development of PML [57]. In these pathological conditions, PML usually develops as a result of exposure to treatment, such as cytotoxic chemotherapy or stem cell transplantation, that significantly suppresses the immunologic system [5].

Although PML was primarily associated with immunocompromised conditions related to hematological malignancies, the number of PML cases substantially increased with the identification of acquired immunodeficiency syndrome (AIDS) in the mid-1980s.

Currently, classic PML is the most common form of the disease, characterized by multifocal white matter lesions in the CNS as a result of the productive lytic infection of oligodendrocytes by JCPyV [57]. Histopathologically, PML shows the triad of demyelination, abnormal astrocytic morphology and oligodendrocytic nuclear inclusions [79]. Although PML symptoms can vary considerably according to the affected brain zone, the most frequent include weakness, sensory deficits, migraines, cognitive dysfunction, aphasia and incoordination [5,6].

Initially, the diagnosis of PML was based on neuropathological findings, requiring the observation of the PML histopathological triad and the detection of JCPyV proteins by immunohistochemistry or DNA by PCR in brain lesions. However, due to the invasiveness of a brain biopsy, PML diagnosis has evolved, accompanying the evolution of diagnostic methods, and is now based on a combination of clinical, radiological and laboratory findings using less-invasive approaches [5,79]. Today, the diagnosis of PML relies on the evaluation of clinical symptoms, brain magnetic resonance imaging (MRI) scans, and the detection of JCPyV DNA in CSF samples through PCR [79].

Brain MRI represents a crucial tool for PML diagnosis. Irregular multifocal lesions, mostly located in parietal, frontal and occipital lobes, characteristic of classic PML, are easily observed in MRI scans. The detection of JCPyV DNA in CSF samples using PCR has led to a significant enhancement in the efficiency of PML diagnosis. This methodology enables the confirmation of JCPyV as the causative agent of CNS disease in an easier, earlier and less-invasive manner [6].

#### 4.1.2. PML–Immune Reconstitution Inflammatory Syndrome

The first cases of PML with associated HIV infection were characterized by a very poor prognosis. At that time, the median survival of PML patients was approximately six months after diagnosis [6]. Nevertheless, the introduction of combination antiretroviral therapy (cART), which improves immune function by suppressing HIV replication, has resulted in prolonged survival and a decline in HIV-associated PML deaths [79].

Although cART has been demonstrated to reverse the stage of immunodeficiency in these PML patients, this process has, in some cases, been associated with severe inflammatory episodes, which is referred to as immune reconstitution inflammatory syndrome (IRIS) [5,57]. Although an inflammatory response during IRIS can mediate JCPyV clearance from the brain of these patients, an extremely exaggerated response can lead to brain damage and the exacerbation of PML neurological symptoms, being potentially life-threatening [81].

The diagnosis of PML-IRIS mainly relies on clinical presentation and brain MRI images. Brain images of PML-IRIS reveal some characteristics that are rarely detected in classic PML cases, such as contrast enhancement, which is associated with edema and mass effect, within PML lesions. Further, the JCPyV genome may not be detected in CSF by PCR techniques [5,82].

#### 4.1.3. Iatrogenic PML

Over the years, the number of PML cases associated with immunomodulatory drugs has been minimal compared to the number of cases associated with HIV infection. However, in the last decade, the expansion of these treatments has led to a rapid increase in associated PML cases [83,84].

New immunomodulatory therapies used in autoimmune disorders such as multiple sclerosis (MS) are associated with an immunosuppression stage that is difficult to reverse. Available data have shown that prolonged treatment with some of these new molecules, such as rituximab, natalizumab, and dimethyl fumarate, is associated with an increased risk of developing PML [6,83,84].

Rituximab is a chimeric immunoglobulin G1 (IgG1) monoclonal antibody specific to CD20 protein that was first approved by the Food and Drug Administration (FDA) in 1997 and later by the European Medicines Agency (EMA) in 1998. In 2002, the first two cases of rituximab-associated PML were reported in non-Hodgkin lymphoma patients, and since then, further cases have been documented [5,80,83,84].

Natalizumab is a humanized monoclonal antibody specific to α_4_ integrin expressed on the surface of B and activated T lymphocytes that was first approved by the FDA in 2004 [85]. In the following year, this drug was withdrawn from the market due to three reported cases of associated PML [57]. However, as natalizumab had been shown to be highly effective in treating MS patients, it was reintroduced in 2005 under a rigorous pharmacovigilance program. Nevertheless, PML cases in natalizumab-treated patients have continued to increase [86].

New immunomodulatory drugs, such as alemtuzumab, have been associated with a lower risk of PML development, potentially offering an alternative therapeutic option to natalizumab [84].

Dimethyl fumarate was approved by the FDA and EMA in 2013. Four cases of PML have been reported with the use of this drug in the treatment of psoriasis, and a further four cases have been reported in fumarate-treated MS patients. In fact, dimethyl fumarate can cause lymphopenia, which increases the risk of developing PML. Thus, the suspension of fumarate treatment should be considered whenever an event of lymphopenia occurs in these patients [84,87].

The diagnosis of PML associated with immunomodulatory drugs mainly relies on the clinical presentation and detection of JCPyV DNA in CSF samples. MRI images obtained in these situations are similar to those obtained in classic PML; nevertheless, in these patients, MRI scans can also be used as prognostic markers, being required every three to four months to detect a possible preclinical stage of PML [6,57].

#### 4.1.4. Other Clinical Manifestations of JCPyV Infection

In addition to oligodendrocytes, JCPyV can also infect granule cells and cortical pyramidal neurons of the CNS. JCPyV infection of granule cells leads to the development of JCPyV granule cell neuronopathy, a disease associated with progressive cerebellar atrophy [88]. Although tropism for these cells mainly occurs for rearranged strains, occasional studies reported the infection with archetype strains [5,88,89], with both archetype and rearranged strains of JCPyV being identified in the CSF of granule cell neuronopathy patients [88].

Cases of fulminant encephalopathy due to JCPyV infection in cortical pyramidal neurons have also been reported, with an archetype variant of JCPyV [5,89].

As these pathologies are very rare, it is difficult to prove the implication of archetype strains in the pathogenesis of such clinical manifestations.

JCPyV-associated meningitis has been suggested due to the detection of the JCPyV genome in the CSF of both immunocompetent and immunocompromised patients with meningeal symptoms. Nevertheless, the prevalence of this condition and its underlying pathogenesis are still largely unknown [3,90].

The diagnosis of JCPyV granule cell neuronopathy, JCPyV encephalopathy and JCPyV meningitis requires the detection of JCPyV DNA in the CSF. Furthermore, a cerebellar biopsy to assess granule cell infection is necessary for the diagnosis of granule cell neuronopathy, and MRI scans are needed for the diagnosis of JCPyV encephalopathy and JCPyV meningitis [5].

The possible association of JCPyV infection with CNS diseases other than PML highlights the importance of including JCPyV in the differential diagnosis of CNS diseases of unknown etiology.

In addition to the implication of JCPyV in CNS diseases, some authors also suggest the possible association of JCPyV with certain types of cancer, including prostate, colorectal and brain cancers [91,92,93]. While some authors have defended this potential association, the incidence of JCPyV in cancerous tissue is highly variable among studies [93,94].

The possible carcinogenic role of JCPyV has been suggested due to the detection of the viral genome and the expression of viral proteins, namely T-antigen, in cancerous tissue. This protein has been demonstrated to bind and inactivate tumor suppressor proteins, such as p53 and pRb, which results in cell cycle dysregulation [91,92,93].

The association of JCPyV with carcinogenesis cannot be established due to the inconsistency of published results, and further studies should be performed.

JCPyV is often disregarded when considering renal pathologies, because BKPyV is the HPyV more frequently associated with such diseases. Nevertheless, as JCPyV establishes a persistent infection in renal cells, immunosuppression related to renal transplantation may induce viral reactivation, resulting in renal JCPyV-associated diseases. In fact, although rare, JCPyV infection has occasionally been associated with nephropathy [95,96,97]. These case reports emphasize the importance of considering JCPyV when diagnosing renal diseases of unknown etiology among immunocompromised individuals.

### 4.2. Therapeutic Strategies

Currently, no effective and proven treatment is available for JCPyV infection. The development of new therapeutic strategies is conditioned by the lack of animal models for PML and the difficulty of formulating antiviral drugs that can cross the blood–brain barrier [57,79]. Until now, two main therapeutic strategies have been explored: direct antiviral therapy; and improvement of the immune system either through reconstituting protective antiviral immunity or reversing immunosuppression [5,6].

Agents with antiviral activity against JCPyV, including the nucleoside analogs cytarabine and cidofovir and the topoisomerase inhibitor topotecan, have been studied. The 5-HT_2A_ receptors have also been proposed as potential therapeutic targets [5]. In some reports, mirtazapine, an atypical antidepressant and 5-HT_2A_ receptor antagonist, has been associated with favorable outcomes in PML patients [98,99]. However, further studies comprising larger population groups are required to confirm the efficacy of this drug in PML treatment [100].

“In vitro” studies have shown that mefloquine, an antimalarial drug, is able to suppress JCPyV replication. Such activity, along with its ability to cross the BBB and reach brain tissue in sufficient concentration to inhibit JCPyV replication, renders mefloquine a promising therapeutic candidate for the treatment of PML. Some reports even suggest that the therapeutic combination of mirtazapine and mefloquine could be an effective treatment for PML patients. However, “in vivo” studies failed to prove the antiviral activity of mefloquine [6], and a previous clinical trial in PML patients was prematurely terminated due to mefloquine’s lack of efficacy [101].

None of the evaluated antiviral therapies were found to effectively reduce neurological symptoms, and the majority of studies were discontinued early due to toxicity or a lack of efficacy [57].

To date, patient survival has mostly depended on the ability to reverse the stage of immunosuppression, as well as on immunotherapy to enhance antiviral immune responses.

In HIV-associated PML patients, the reconstitution of protective antiviral immunity can be achieved by initiating cART. In MS patients, such reconstitution may be achieved by discontinuing immunotherapies [57].

Other approaches have also been proposed, including treatment with interleukin-7 (IL-7) and IL-5 [102,103]. Human recombinant cytokines may be promising strategies for boosting effector T cell responses against JCPyV [86,104]. In fact, the administration of IL-5 was associated with improved prognosis in some PML patients, particularly through the stimulation of natural killer (NK) cells and CD8+ T cells, which may favor JCPyV clearance [86].

IL-7 plays a crucial role in T cell development and mature T cell homeostasis [86,104]. The use of IL-7 in a limited number of PML patients with lymphopenia showed promising outcomes by increasing CD4+ T cell counts and specific immune responses against JCPyV. For instance, Sospedra and colleagues (2014) treated two PML patients with IL-7 along with a therapeutic vaccine composed of JCPyV VP1 protein. This therapeutic strategy allowed the clinical stabilization of the patients by increasing VP1-specific CD4+ T cell responses and restoring the immune system [103,105].

Using highly conserved VP1 protein sequences, Kanse and colleagues (2023) designed a multi-epitope that stimulates immune response against JCPyV. The design of a multi-epitope approach aimed to improve immunogenicity and expand the coverage of antigenic variants. Through this approach, the risk of viral escape of certain variants is reduced, and a more comprehensive protection is obtained. Despite the lack of experimental validation, the stimulation of the immune system showed a robust response, and the obtained data may have implications in the development of a vaccine against JCPyV [106].

Checkpoint inhibitors, normally reserved for cancer treatment, have been tested to improve the immune response to JCPyV. Persistent viral infection is usually associated with immune exhaustion and loss of efficacy of the immune response, which, in turn, can compromise viral clearance. Therefore, checkpoint inhibitor treatment is used to reinvigorate antiviral immune responses by stimulating effector lymphocytes [6]. The blockade of checkpoint molecules, such as programmed cell death protein 1 (PD-1), expressed on the surface of CD4+ and CD8+ T cells from the blood and CSF of PML patients, has been shown to increase cellular immune responses against JCPyV [107]. In some cases, the use of nivolumab and pembrolizumab, anti-PD-1 drugs, was shown to enhance JCPyV-specific activity in both CD4+ and CD8+ T cells, leading to clinical and radiological benefits [108,109]. However, these data are based on limited case reports, and adverse events are well documented in oncology applications [6,109].

The use of JCPyV-specific T cells has also been reported [109,110,111]. A 19-year-old patient with PML and profound immunosuppression was treated using donor-derived JCPyV-specific T cells that had been generated “in vitro”. Following treatment, the patient exhibited a significant recovery, with JCPyV not detected in the CSF and no adverse effects observed [110,112,113].

Although promising results have been described for these therapeutic strategies, more studies are required to confirm their safety and effectiveness.

## 5. Conclusions

JCPyV is a ubiquitous human virus responsible for severe diseases of the CNS in immunocompromised patients, including those treated with immunomodulatory drugs such as natalizumab [17]. The most common JCPyV-associated pathology is PML, an often-fatal demyelinating disease. Despite the severity of these conditions, no specific treatment or vaccine to prevent JCPyV infection is available [4]. Thus, to prevent infection in risk groups, such as natalizumab-treated patients, avoiding transmission may be an important strategy to prevent JCPyV-associated diseases.

Nevertheless, the main mode of JCPyV transmission remains to be defined. Seroprevalence data and recent studies point to the implication of different modes of transmission throughout life. Respiratory transmission appears to be an important route among young children [18]. In young adults, vaginal secretions and semen may be implicated in JCPyV transmission, probably through the sexual route [23,24,25]. The fecal–oral route should also be considered, because the JCPyV genome has been frequently detected in treated wastewater discharged into the environment, which may contribute to the viral dissemination and contamination of bivalves, as well as agricultural products irrigated with these waters, including fresh vegetables and soft fruits [21,22]. Thus, acknowledging the transmission routes of JCPyV is crucial for implementing protective measures and preventing infection in high-risk groups, such as patients treated with natalizumab, while no effective treatment or vaccine is available.

Further studies are required to clarify the routes of JCPyV transmission, understand its pathogenesis, and support the development of preventive and therapeutic strategies.

## Data Availability

No new data were created or analyzed in this study.
